# Puffing Topography and Nicotine Intake of Electronic Cigarette Users

**DOI:** 10.1371/journal.pone.0117222

**Published:** 2015-02-09

**Authors:** Rachel Z. Behar, My Hua, Prue Talbot

**Affiliations:** 1 Cell Molecular and Developmental Biology Graduate Program, University of California Riverside, Riverside, California, United States of America; 2 Department of Cell Biology and Neuroscience, University of California Riverside, Riverside, California, United States of America; The University of Auckland, NEW ZEALAND

## Abstract

**Background:**

Prior electronic cigarette (EC) topography data are based on two video analyses with limited parameters. Alternate methods for measuring topography are needed to understand EC use and nicotine intake.

**Objectives:**

This study evaluated EC topography with a CReSS Pocket device and quantified nicotine intake.

**Methods:**

Validation tests on pressure drop, flow rate, and volume confirmed reliable performance of the CReSS Pocket device. Twenty participants used Blu Cigs and V2 Cigs for 10 minute intervals with a 10–15 minute break between brands. Brand order was reversed and repeated within 7 days Data were analyzed to determine puff duration, puff count, volume, flow rate, peak flow, and inter-puff interval. Nicotine intake was estimated from cartomizer fluid consumption and topography data.

**Results:**

Nine patterns of EC use were identified. The average puff count and inter-puff interval were 32 puffs and 17.9 seconds. All participants, except one, took more than 20 puffs/10 minutes. The averages for puff duration (2.65 seconds/puff), volume/puff (51ml/puff), total puff volume (1,579 ml), EC fluid consumption (79.6 mg), flow rate (20 ml/s), and peak flow rate (27 ml/s) were determined for 10-minute sessions. All parameters except total puff count were significantly different for Blu versus V2 EC. Total volume for Blu versus V2 was four-times higher than for conventional cigarettes. Average nicotine intake for Blu and V2 across both sessions was 1.2 ± 0.5 mg and 1.4 ± 0.7 mg, respectively, which is similar to conventional smokers.

**Conclusions:**

EC puffing topography was variable among participants in the study, but often similar within an individual between brands or days. Puff duration, inter-puff interval, and puff volume varied from conventional cigarette standards. Data on total puff volume and nicotine intake are consistent with compensatory usage of EC. These data can contribute to the development of a standard protocol for laboratory testing of EC products.

## Introduction

Electronic cigarettes (EC) are nicotine delivery products that are rapidly becoming popular worldwide [1, 2]. EC deliver aerosol to users by heating a humectant that generally consists of propylene glycol or vegetable glycerin, artificial flavorings, and varying concentrations of nicotine [3, 4]. Users have the option of purchasing prepackaged cartridges and cartomizers with different nicotine concentrations or refill fluid bottles with varying flavors and nicotine concentrations. EC vary widely in their performance with regards to pressure drop, the airflow rate that activates the battery, delivery of aerosol, and the number of puffs produced per cartomizer [3–6]. Discrepancies between labeled nicotine concentrations and actual concentrations have also been reported in bottles of refill fluid [7–9]. While the FDA has recently issued deeming regulations for EC [10], the manufacture and marketing of EC and their refill fluids are currently not regulated, and these products are readily available for purchase in stores and online. Although EC contain lower levels of carcinogens than conventional cigarettes and may therefore be considered a harm reduction product [11, 12], newer models with variable battery power produce elevated levels of carbonyls, including the carcinogens formaldehyde and acetaldehyde [13, 14]. While the long-term health effects of EC are not yet known, various adverse outcomes have been reported by short-term users [15–18] and *in vitro* testing has shown cytotoxicity associated with some products and flavors, including cinnamaldehyde [19, 20]. Because EC are designed to be used repeatedly, they can easily be used for long durations of time/session, which could also contribute to increased exposure levels and adverse health effects.

Information on EC topography can provide insight into puffing behavior, potential nicotine intake, as well as variability or unique patterns of usage between different brands, models, and users. In addition, topography data are needed to understand baseline characteristics pertaining to EC use, which can then be used to establish standardized smoking machine protocols. Such standards would be valuable to research labs working with these products.

While conventional cigarette topography has been well documented [21], relatively little is known about the topography of EC users [22]. In data mined from YouTube videos, the average puff duration for EC users was 4.3 seconds, which is nearly twice the duration of conventional smokers [23]. A subsequent video study, that recorded EC use during 20 minutes sessions, confirmed these results on puff duration and extended observations to other topography parameters [24]. While video analyses are useful, they are limited in the number of parameters that can be obtained, and other methods are needed to evaluate EC topography. The Clinical Research Support System (CReSS Pocket) is a handheld device that measures parameters such as puff duration, puff count, total volume, flow rate, peak flow rate, and intervals between puffs. The CReSS has been used previously to evaluate topography of conventional cigarettes users [21, 25]. In this study, we first validated the CReSS Pocket for EC use. We then evaluated EC topography parameters using two EC brands and used the cartomizer fluid consumed and puff count to calculate nicotine intake for each user and each brand.

## Materials and Methods

### EC Purchase

Two brands of EC (Blu Cigs and V2 Cigs) were purchased with their starter kits on the Internet. These brands were chosen as they are top sellers and readily available in shops and over the Internet. Tobacco-flavored cartomizers with label concentrations of 16 mg/ml (Blu Cigs) and 18 mg/ml (V2 Cigs) of nicotine were used. Each participant was provided with a cartomizer, which was stored in a 15 ml conical tube (BD Falcon) after use. All EC cartomizers were inventoried and tracked using a letter system labeled onto individual packs (e.g., Carton A = CA, etc.) and a code to identify the cartomizer position in the pack (e.g., CA A1). After participants completed their first day’s session, individual cartomizers and disposable mouthpieces were stored until participants came back for their second day of the study.

### CReSS Pocket Device

A cigarette topography device, the Clinical Research Support System (CReSS Pocket, version 3, Borgwaldt KC), was purchased with an EC adaptor. However, no manual for EC use was provided by the company. Previously, the CReSS Pocket has been used to study conventional cigarette topography [21]. Its adaption for use with EC topography is new, and 4–5 months were required to learn how to operate the CReSS Pocket reliably with EC users.

### CReSS Pocket Calibration and Cleaning

The CReSS Pocket device was calibrated before each use in each session with CReSS software running on a PC desktop computer. The EC cartomizer without its battery was inserted into the CReSS Pocket as advised by Borgwaldt KC Tech Support. A syringe with tubing was attached to the other end of the cartomizer, the syringe was plunged manually to approximately 40 ml, and the CReSS Pocket software calculated volume of the “puff”. All CReSS volume recordings that were +/-3 ml of the syringe reading were deemed acceptable values for calibration. When volumes computed by the CReSS software exceeded +/-3 ml of the syringe volume, recalibration was done. The CReSS Pocket was periodically cleaned by taking off the mouthpiece and removing any EC fluid or residue that was present.

### CReSS Pocket Validation

#### Pressure Drop

A smoking machine (University of Kentucky, Lexington, KY) was connected to a U-shaped water manometer and MasterFlex peristaltic pump (Barnart Company, Barrington, IL, Model #7520–00) by Cole Parmer MasterFlex Tygon tubing [4, 26, 27]. The smoking machine drew 4.3 second puffs at 60 second intervals. The water manometer measured pressure drop (mmH_2_O) across both EC brands during each puff with and without the CReSS Pocket during four trials to determine if the CReSS Pocket affected pressure drop.

#### Flow Rate

The smoking machine described above was used to validate the flow rate measurements that the CReSS Pocket recorded. Both brands of EC were manually tested for three trials each at three different flow rates (19 ml/s, 21 ml/s, and 24 ml/s). The peak flow rates for each of the trials recorded by the CReSS Pocket were averaged and compared to the flow rate when the CReSS Pocket was not connected to the machine.

#### Volume

A 60 ml handheld syringe with tubing at one end was attached to the mouthpiece end of the CReSS Pocket to validate volume measurements. Three trials were performed in which the syringe was manually pulled to approximately 40 ml, and the CReSS Pocket recorded the volume measurement each time. The average volume recorded on the syringe was compared to the average volume measured by the CReSS Pocket.

### Participant Recruitment

An advertisement was sent through the electronic mail system at the University of California, Riverside to recruit participants for the study. To be eligible, participants had to be: (1) 18 years of age or older, (2) experienced EC users (three months or more), (3) accustomed to using EC with 12 mg of nicotine/ml or higher concentrations, and (4) in overall good health with no pre-existing health conditions. Applicants were excluded if: (1) they were pregnant, (2) used EC without nicotine, (3) were not in optimal health the first day of arrival (i.e. sick/flu), (4) were not able to return for their second day within a 7 day time period, (5) they drew fluid into the CReSS Pocket during puffing, or (6) they chose to withdraw from the study. A pool of 20 EC users who met the above inclusion/exclusion criteria was studied.

### Experimental Design

Each participant was supplied with a fresh Blu and V2 cartomizer. Cartomizers were primed with a 60 ml syringe before participants used them in order to prevent EC fluid from leaking out of the cartomizer into the CReSS Pocket. For priming, an EC was connected through tubing to a 60 ml syringe and plunged 10 times to simulate puffs taken by a user. After each puff, aerosol that was collected in the syringe was expelled under a fume hood.

Participants were involved in the study for 2 days within a 1-week period and were asked to abstain from EC or conventional cigarette use for at least 1 hour before arrival. On the first day, each participant signed informed consent paperwork, approved by the UCR Human Research Review Board, and filled out a questionnaire on their smoking and EC history.

The participants were escorted to a designated outdoor study site and were observed at the start of each session for 1–3 puffs on either a Blu or V2 EC to ensure the EC and CReSS Pocket worked properly. Participants were then left alone and used the EC *ad libitum* for 10 minutes, followed by a 10–15 minute break, after which they used the second brand for 10 minutes. After the first 10-minute session, individual user data were uploaded to a desktop computer with the CReSS Pocket software before recalibrating the device for the second EC brand. Data for the second brand were immediately transferred after collection from the CReSS pocket to the desktop computer.

On the first day of their sessions, participants were assigned to use Blu or V2 in alternating sequence depending on what brand the previous participant used first. On the second day, which occurred within 7 days of the first session, the same procedures were followed except the brand order was reversed for each participant. Cartomizers were weighed after priming and again after completion of the sessions on the first and second day for each participant.

### Determining Nicotine Concentration of Each Brand

High performance liquid chromatography (HPLC) was performed on a randomly selected Blu and V2 cartomizer to determine the nicotine concentration as described in detail previously [19]. HPLC analysis showed the selected Blu cartomizer that was labeled 16 mg of nicotine/ml contained 16.324 mg/ml, while the V2 cartomizer, which was labeled 18 mg of nicotine/ml, contained 15.612 mg/ml. The nicotine concentrations specified on the labels of the two brands were used in subsequent calculations to determine the total nicotine concentration/puff for each participant in the study.

### Calculating Nicotine Intake/Session

To determine nicotine intake, the following 7 steps were performed. (1) Cartomizer weights, which were recorded before and after each study session, were subtracted to find the total weight of EC fluid consumed by each user. (2) The fluid densities [Blu (1.145 g/ml) and V2 (1.099 g/ml)] were determined by taking the average weight of the fluid and dividing it by the average weight of water. (3) The total weight consumed was divided by the fluid density to find the volume consumed. (4) Volume/puff was then calculated by dividing the volume consumed by the puff count for that session. (5) To determine the nicotine concentration consumed in weight/puff, the volume/puff was multiplied by the concentration of nicotine given on the label. (6) Total nicotine consumed in a session was calculated by multiplying the nicotine concentration in weight/puff by the puff count for that session. (7) Finally these values were converted from g to mg.

### Statistical Analysis

Paired t-tests were used to compare Blu Day 1 vs. Blu Day 2, V2 Day 1 vs. V2 Day 2, and Combined Blu vs. Combined V2, while unpaired t-tests were used to compare dual and EC users. Additional paired t-test comparisons were made between Blu Day 1 vs. V2 Day 1, Blu Day 2 vs. V2 Day 2. All analyses were done using GraphPad Prism software (GraphPad, San Diego, California, USA). Means were considered significantly different when p < 0.05.

## Results

### Characteristics of the Study Participants

Twenty participants (16 males and 4 females) were qualified for inclusion in the EC puffing topography study (Table 1). Most participants were between the ages of 18–25 (N = 19), and most (N = 17) had experience with conventional cigarettes. All participants began smoking conventional cigarettes as teenagers. Those who smoked cigarettes generally used one pack or less/day (N = 15). At the time they entered the study, eight participants used both EC and conventional cigarettes (dual users), and seven of those smoked less than half a pack of conventional cigarettes/day.

**Table 1:** Demographics of EC Participants, Smoking History, and EC Use.

**I. BASIC PARTICIPANT INFORMATION**		**Responses**
A. Age Range		
	18–25	19
	26–40	1
B. Gender		
	Male	16
	Female	4
C. Dual Use of EC and Conventional Cigarettes		
	No	8
	Yes	12
*If yes*, *how many packs while using EC?*		
	<0.5 pack	7
	0.5–1 pack	1
**II. CONVENTIONAL SMOKING HISTORY**		
A. Experience using conventional cigarettes		
	Yes	17
	No	3
*If yes*, *for how long?*		
	0–2 years	7
	3–4 years	4
	5+ years	5
	Infrequent User/One time Smoker	1
*If yes*, *starting at what age?*		
	<15	3
	15–16	8
	17–18	6
*If yes*, *how many packs per day?*		
	<0.5 pack	8
	0.5–1 pack	7
	2 packs+	1
	Did not state	1
**III. EC USAGE HISTORY**		
A. Length of EC History Use		
	3–11 months	7
	1–2 years	10
	3–4 years	3
B. User estimated puffs/day for EC		
	1–100	12
	101–200	6
	201–300	1
	Did not state	1
C. Estimated times of EC use per day		
	0–10	16
	11–20	3
	21+	1
D. Estimated duration (in minutes) for each use		
	0–9 minutes	7
	10–30 minutes	12
	30 minutes—1 hour+	1
E. Style of EC Used		
	Cartomizer/Automatic Button	3
	Disposable	1
	Mini EC	1
	Tank	10
	Vapor Pen	2
	Other	3
F. What nicotine concentration(s) do you use?*		
	0 mg	1
	1–10 mg	7
	11–18 mg	14
	18+ mg	2
		

* denotes a category where users reported multiple answers.

Most participants had used EC for more than a year (N = 13), and the majority estimated that they puffed EC up to 200 times/day. Users had experience with various styles of EC with tank models being used by half of the participants. Some participants had experience using more than one concentration of nicotine, which is why the total number of responses for this section exceeded the number of participants. Within this category, 11–18 mg of nicotine/ml was the most popular concentration.

### Validation of the CReSS Pocket for EC Use

**Figure 1:**
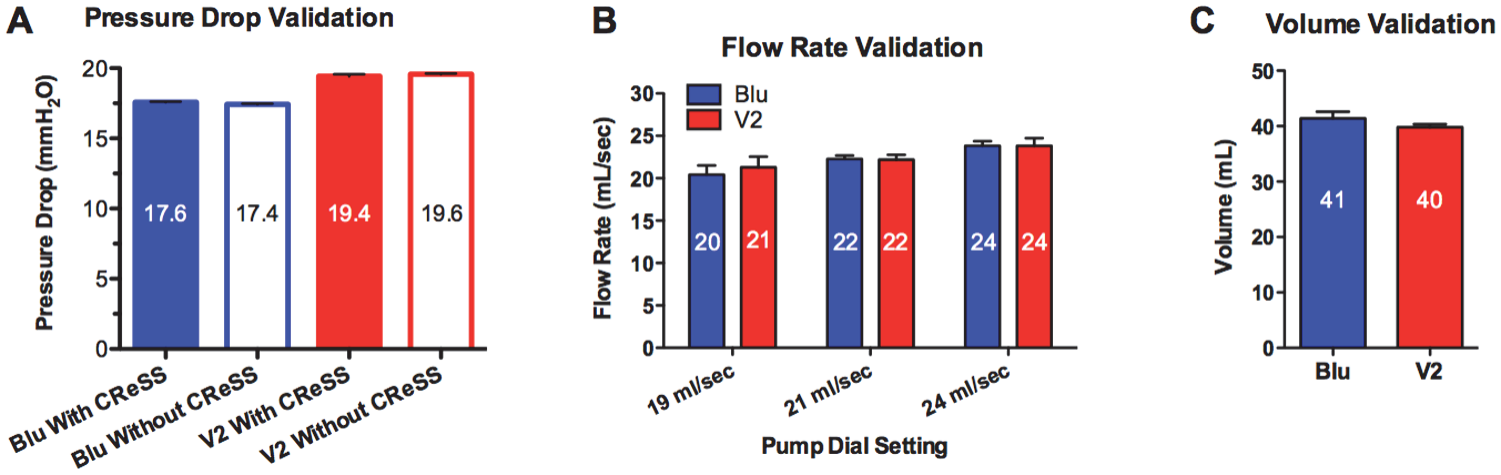
CReSS Pocket Validation Tests. (A) Pressure drop validation tests for each brand with and without the CReSS Pocket connected to the smoking machine (n = 4). (B) Flow rate validation tests for each brand connected to the CReSS Pocket puffed at three different flow rates using a smoking machine (n = 3). (C) Volume/puff validation tests for each brand performed by manually pulling a 60 ml syringe to approximately 40 ml (n = 3). For all validation tests, values are the means ± SD. In Fig. 1A, SD are small and do not show well.

Validation tests were performed to determine if the CReSS Pocket accurately measured pressure drop, flow rate, and volume (Fig. 1). There was a negligible (1%) difference in pressure drop for both brands when the CReSS Pocket was connected to the smoking machine (Fig. 1A). For the flow rate and volume validation tests, the CReSS Pocket recorded values within 10% of the expected values (Fig. 1B, C). There were no significant differences between measurements with and without the CReSS. All validation tests were considered acceptable.

### Puffing Topography for the Population of EC Users

In Table 2, the average puff count, puff duration, total volume, volume/puff, flow rate, peak flow rate, and inter-puff interval are shown for each session on both days for Blu and V2. Combined data for each of the brands and overall combined data across all sessions are also shown.

**Table 2:** Summary of Topography Data.

	**Blu Day 1**	**Blu Day 2**	**V2 Day 1**	**V2 Day 2**	**Blu Combined**	**V2 Combined**	**All Combined**	**Dual User**	**EC Only User**
**Avg Puff Count**	33±10	32±10	30 ±9	32±10	33 ±8	31±8	32±8	32±7	32±9
**Avg Puff Duration (s)**	2.82 ±1.02	2.68 ±0.98	2.62 ±1.13	2.46 ±0.99	**2.75±0.96** ^a^ *	**2.54±1.04** ^a^ *	2.65±0.98	2.84 ±0.93	2.52 ±1.07
**Avg Total Volume (ml)**	1934 ±841	1773 ±884	1318 ±498	1290 ±560	**1853±786** ^b^ ***	**1304±477** ^b^ ***	1579±599	1808 ±701	1426 ±761
**Avg Volume/Puff (ml)**	58±23	54±22	47±24	43±22	**56±22** ^c^ ***	**45±22** ^c^ ***	51±21	59±25	45 ±20
**Avg Flow Rate (ml/s)**	21±7	21±6	19±6	18±7	**21±6** ^d^ ***	**18±6** ^d^ ***	20±6	21±6	19±7
**Avg Peak Flow Rate (ml/s)**	28±11	29 ±9	25 ±9	24±10	**29±10** ^e^ ***	**25±9** ^e^ ***	27±9	29±9	25±10
**Avg Inter-Puff Interval (s)**	16.2 ±7.8	17.4 ±10.2	19.0 ±7.0	18.9 ±10.3	**16.9±8.2** ^f^ *	**18.9±7.3** ^f^ *	17.9±7.5	17.3 ±6.6	18.3 ±10.2

Data are presented as means ± standard deviations. Blu Day 1 vs Blu Day 2, V2 Day 1 vs V2 Day 2, and Blu Combined vs V2 Combined were analyzed using two-tailed paired t-tests.

Dual Users and EC Only Users were analyzed using two-tailed unpaired t-tests. Pairs that were compared and were significantly different are shown by superscript letters and are in bold.

* = p<0.05;

*** = p<0.001.

Avg = Average.

Statistical analyses were performed using a two-tailed paired t-test to compare Blu on Day 1 and Day 2 as well as V2 on Day 1 and Day 2. Because no significant differences were found for any of the parameters in these comparisons, data for each brand were combined and analyzed using paired t-tests. The combined data for Blu were significantly different from the combined data for V2 for all parameters except average puff count (Table 2), indicating that puffing topography was different for the two brands of EC.

Data across both brands and both days were combined to give overall measurements of each parameter for EC use (Table 2, All Combined). The average puff volume (51 ±21 ml), average puff duration (2.65 ±0.98 seconds), and average inter-puff interval (17.9 ±7.5 seconds) were determined from the combined data.

‘Dual user’ (N = 8) and ‘EC only user’ (N = 12) data, analyzed by unpaired t-tests, did not differ significantly for any of the topography parameters in Table 2, although dual users had higher volumes, flow rates, and puff duration than EC only users. EC topography between these categories of users also showed high variance, which may have masked significance.

**Figure 2:**
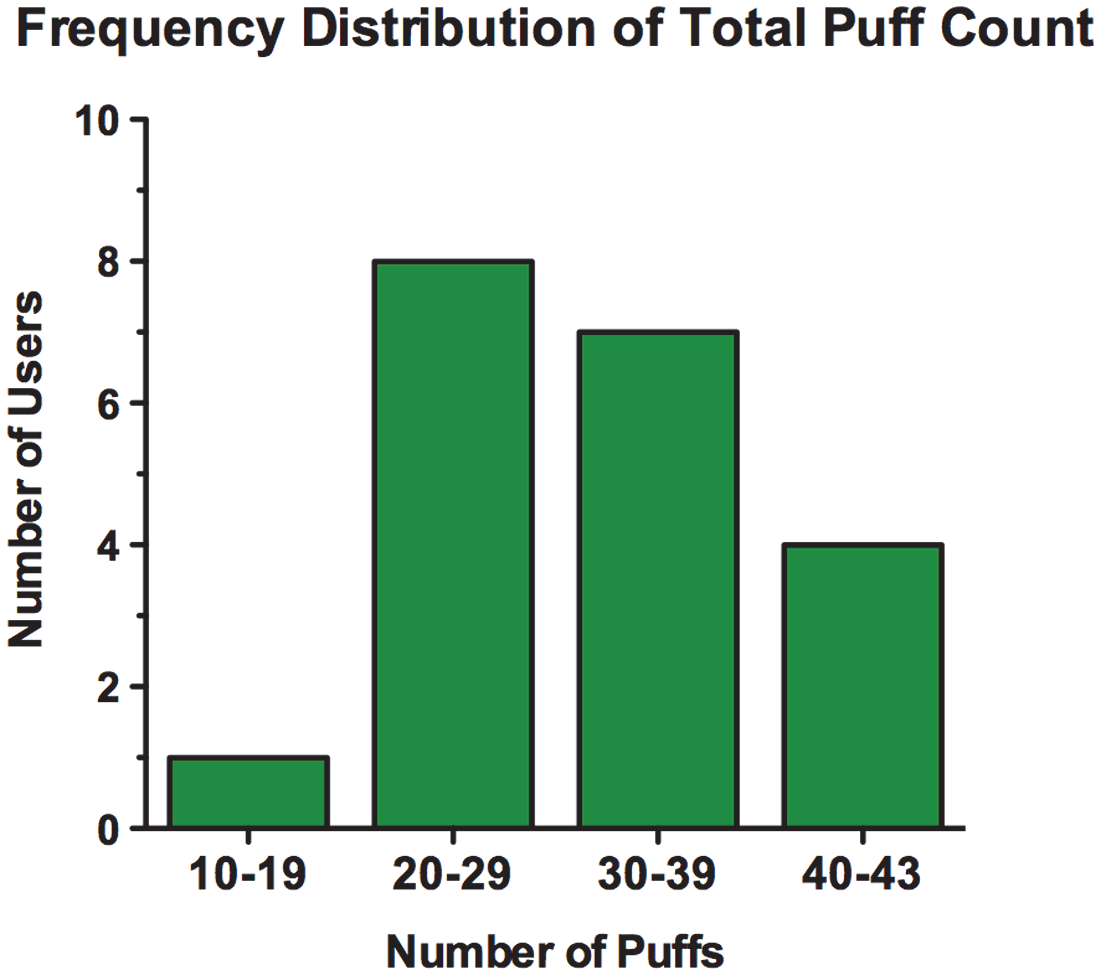
Frequency distribution of puff count for all users. Frequency distribution of total puff counts for all users broken down into different puff number intervals (N = 20).

The total number of puffs/10 minutes ranged from 13 to 42. As shown in the frequency graph (Fig. 2), most users took between 20–39 puffs/10 minutes, while fewer users took 40–43 puffs, and only one user took between 10–19 puffs.

### EC Topography for Individual Users

**Figure 3:**
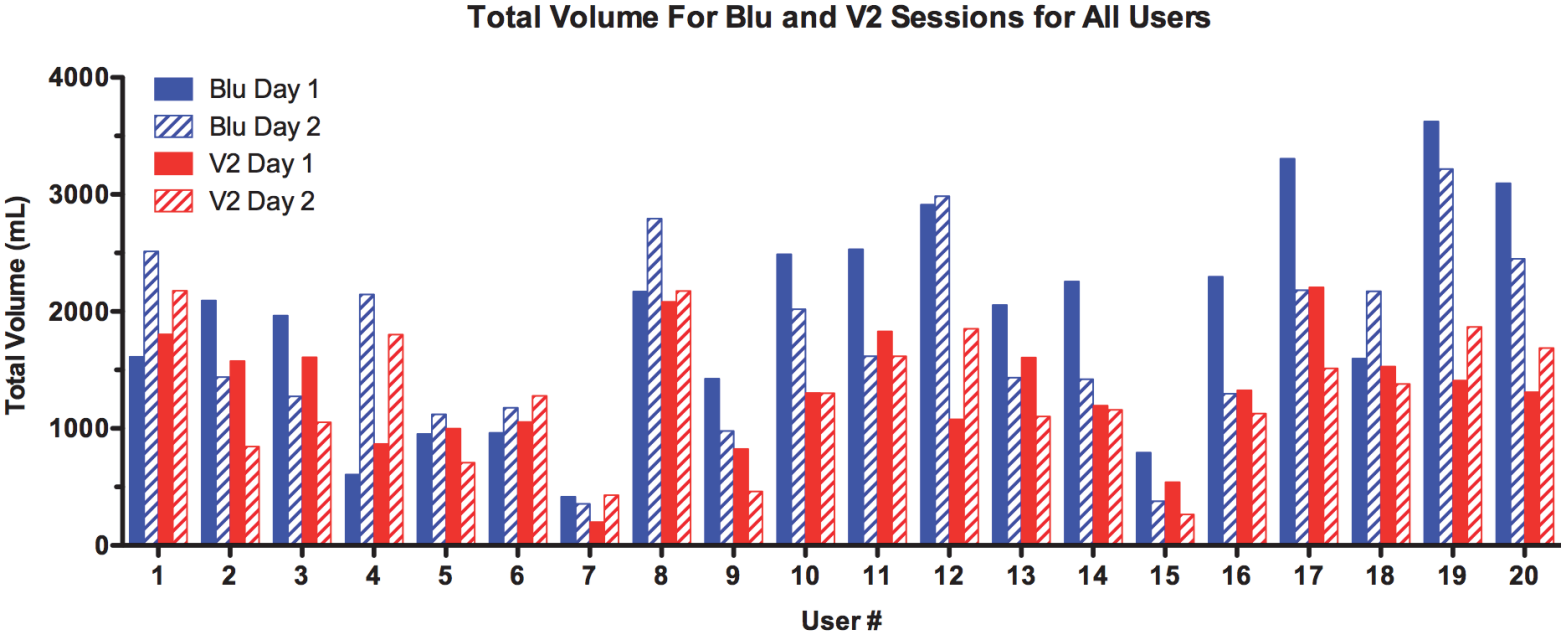
Total volumes for all user sessions. Total volume for each session of Blu and V2 on Day 1 and 2 for all users (N = 20).

In general, the total volume/10 minute session varied among users (Fig. 3). Some participants maintained consistency in total volume across both days and both brands (e.g. # 5, 6, and 8), but often this was not the case. Additionally, the total volume for Blu generally exceeded that for V2 (Fig. 3).

**Figure 4:**
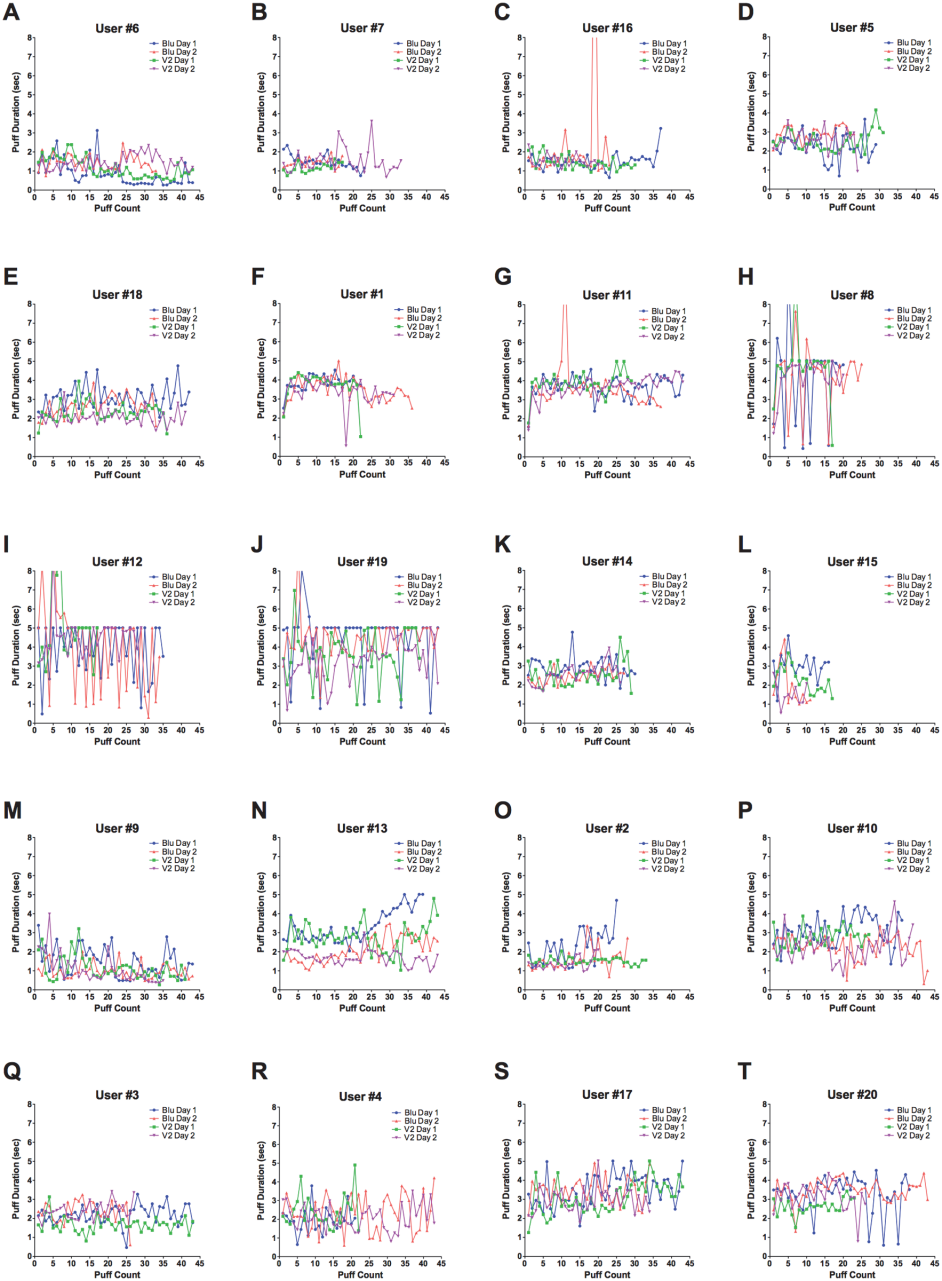
Nine topography patterns of EC use were detected for puff count vs puff duration. (A-C) are users who had consistently low puff durations. (D, E) are users with midrange puff durations. (F, G) are users with high puff durations. (H-J) are users with erratic puff durations across all sessions. (K, L) are users who consistently had fewer total puffs. (M, N) are users with consistently higher total puff counts. (O, P) are users who varied puff counts across all sessions. (Q-S) are users who had different puff counts on Day 1 and Day 2. (T) is a user who puffed Blu more times than V2.

Nine patterns of EC use were identified when puff duration was plotted as a function of time (Fig. 4). Some users had consistently low (Fig. 4A–C), midrange (Fig. 4D, E), or high puff (Fig. 4F, G) durations. Other users had erratic puff durations across all sessions for both brands, indicating they took a small pre-puff before a main puff (Fig. 4H–J). Other patterns showed that some users had consistently fewer (Fig. 4K, L) or higher (Fig. 4M, N) total puff counts or they varied in total puff count across all sessions for both brands (Fig. 4O, P). Additionally, some users varied their puff count from day to day (Fig. 4Q–S), and one user altered their puff count between the different brands (Fig. 4T). Although nine distinct patterns of use were identified from the 20 participant datasets, an individual user could be categorized into two or more patterns of use. There was no association between dual or EC only users and any particular pattern of use. These data demonstrate that patterns of use vary in the population, but individual users can show consistency of use among sessions, days, and brands.

### Fluid consumption

Fluid consumption varied among users. The weight of the EC fluid consumed was recorded after each 10-minute session. For Blu, fluid consumption weights ranged from 9.3 mg to 146.5 mg (mean = 78.5 mg ± 32.2), while for V2, the range was 7.5 to 171.4 mg (mean = 80.6 mg ± 45.7). There was no significant difference between fluid consumed for Blu or V2. The average values for the combined days across both brands ranged from 11.0 mg to 129.6 mg (mean = 79.6 mg ± 36.2).

### Nicotine Intake

Table 3 summarizes the amount of nicotine intake for Blu and V2 users after each session. Both the nicotine concentrations on the labels and the actual concentrations determined by HPLC are presented. The average weights of nicotine consumed for Blu Day 1 and Blu Day 2 were similar to V2 Day 1 and V2 Day 2. After combining both days, the averages for Blu and V2 were significantly different (p < 0.05), but this small difference may not be scientifically important. EC participants’ nicotine intake ranged from 0.2–2.6 mg/session (labeled nicotine concentration) and 0.2–2.3 mg/session (HPLC determined nicotine concentration) for both brands. On average, dual users had higher nicotine uptake for both Blu and V2 Combined data from both sessions as well as for the Combined Total for both brands.

**Table 3:** Total Nicotine Concentration Consumed (weight in mg/10 minute session).

	**Day 1 (mg)**	**Day 2 (mg)**	**Combined Days (mg)**
**Blu (N = 20)**			
16 mg^a^	1.1 ± 0.5	1.2 ± 0.6	1.2 ± 0.5^c^*
16.324 mg^b^	1.1 ± 0.5	1.2 ± 0.6	1.2 ± 0.5
Range 16 mg^a^	0.2–2.0	0.1–2.6	0.2–1.9
Range 16.324 mg^b^	0.2–2.1	0.1–2.6	0.2–1.9
			
**V2 (N = 20)**			
18 mg^a^	1.2 ± 0.7	1.5 ± 0.9	1.4 ± 0.7^c^*
15.612 mg^b^	1.1 ± 0.6	1.3 ± 0.7	1.2 ± 0.6
Range 18 mg^a^	0.1–2.8	0.3–3.3	0.2–2.6
Range 15.612 mg^b^	0.1–2.4	0.2–2.8	0.2–2.3
			
	**Blu Combined (mg)**	**V2 Combined (mg)**	**Combined Total (mg)**
**Dual Users (N = 8)**	1.2 ± 0.3	1.6 ± 0.4	1.4 ± 0.3
**EC Only Users (N = 12)**	1.1 ± 0.5	1.2 ± 0.8	1.1 ± 0.6

Data are presented as means ± standard deviations. Ranges depict lowest to highest nicotine concentrations consumed by participants across both brands and are shown in percentage (%).

^a^ = Nicotine concentration on label of EC.

^b^ = Nicotine concentration determined by HPLC. Blu Day 1 vs V2 Day1, Blu Day2 vs V2 Day 2, and Combined Blu vs Combined V2 were analyzed using paired t-tests. Comparisons were done using groups with labeled nicotine concentrations. The pair that was significantly different is shown by

^c^ and is in bold.

* = p< 0.05. Dual Users and EC Only Users were analyzed using two-tailed unpaired t-tests.

**Figure 5:**
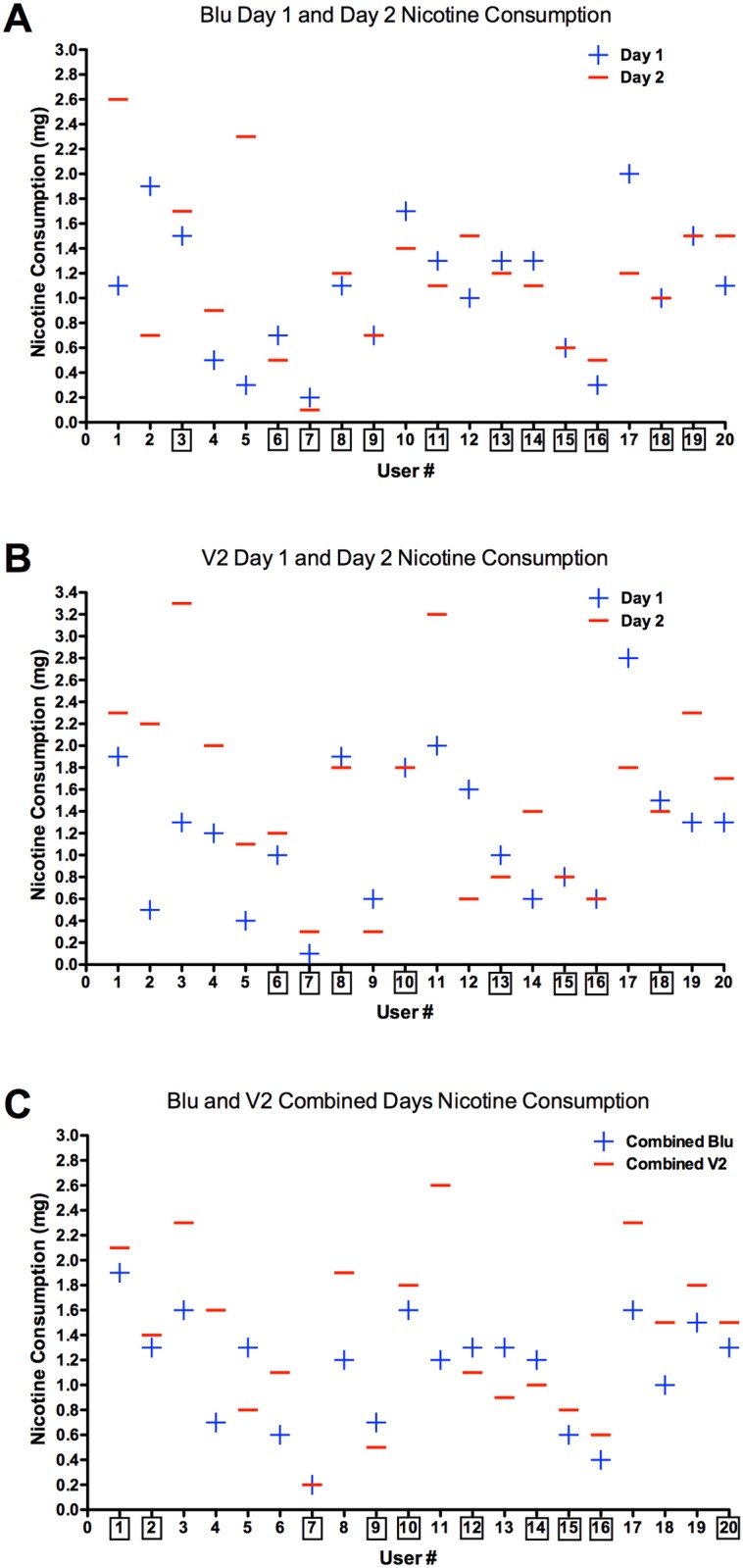
Nicotine intake for all users. (A) Nicotine intake for all users for Blu on Day 1 and Day 2. (B) Nicotine intake for all users for V2 on Day 1 and Day 2. (C) Nicotine intake for all users with Blu Days 1 and 2 combined and V2 Days 1 and 2 combined. Black boxes around user numbers indicate a nicotine intake difference that was ≤ 0.2mg, which was considered to be similar nicotine intake from day to day or brand to brand.

EC participants whose nicotine consumption on Days 1 and 2 differed by ≤ 0.2 mg were considered to have similar nicotine intake on consecutive days (e.g., User 8 Fig. 5A and B), Twelve of the 20 EC participants had similar nicotine intake for Blu Day 1 and Day 2, while eight of 20 EC participants had similar nicotine intake for V2 Day 1 and Day 2 (Fig. 5A and B). When data for Days 1 and 2 were combined for each brand of cigarette, 10 of 20 participants consumed similar amounts of nicotine for both brands (Fig. 5C).

## Discussion

Puffing topography data increase the understanding of EC user behavior and exposure and are needed to facilitate the development of a standard protocol for EC testing in laboratory and clinical studies. This is the first study to validate the CReSS Pocket for measuring EC puffing topography and to collect data on a full range of topography parameters. While the CReSS Pocket had limitations, it was possible to evaluate puffing topography for 20 experienced users in a longitudinal cross over design. A previous study with conventional cigarettes compared data collected with a similar CReSS device to that collected by direct observation and found each method to be reliable [25]. A second study showed that the reliability of topography devices, such as the CReSS, is not affected by the attachment of a mouthpiece adapter [28], which is in agreement with our study. Adding an EC mouthpiece to the CReSS Pocket did not alter its performance, as shown by our validation measurements.

The data in Table 2 show that puffing topography varied significantly with the type of EC used. All parameters, except total puff count, were significantly different for Blu and V2. For all parameters that were different, except average inter puff interval, values for Blu were higher than for V2. These difference may be related to EC performance which is likewise highly variable among brands [4–6] and to the possibility that nicotine delivery may have been slightly more efficient with V2 (Table 3). Differences in topography between brands will need to be considered when establishing a standard protocol for EC evaluations.

EC puffing topography was also highly variable among users, which is consistent with conventional cigarette user data showing high variability between individuals [29]. When analyzing individual EC user puff duration vs. puff count, nine patterns were identified. For example, one user puffed less than 20 puffs/session, whereas some users took more than 40 puffs/session. Puff durations were likewise highly variable within the population. Comparisons between dual users and EC only users found no significance between these groups in any puffing topography parameters or nicotine intake. This may be due to the relatively small population size and high variance in our study. Additionally, 15% of the EC participants in our study had no previous history of conventional cigarette use (Table 1). For these participants, EC were a gateway product that introduced them to nicotine, which could lead to nicotine dependence [30–33]. Although these users had never previously smoked conventional cigarettes, there was no evidence that they used EC any differently than others in the study group.

In spite of differences in topography between EC users, individual participants often showed remarkable consistency in multiple areas of EC puffing topography. For example, individual users often had consistent puff duration or puff counts across sessions (e.g. User #9 and #14). Many participants also drew in similar total volumes across both sessions regardless of brands. EC participants who displayed a “pre-puffing” behavior were often likely to exhibit this behavior on both days for both brands. Additionally, 50% of the users displayed consistent nicotine intake between brands and 40–60% of users displayed similar nicotine intake between days. Like EC users, individual conventional cigarette smokers often have stable smoking behaviors over time [29].

The nicotine intake was calculated based on the weight of nicotine (mg) consumed. The combined averages for nicotine intake [1.2 ± 0.5 mg/10 minute (Blu) and 1.4 ± 0.7 mg/10 minute (V2) (Table 3)] are similar to, but higher, than in a previous study in which nicotine intake was 0.46 ± 0.12 mg per 5 minutes and 1.63 ± 0.41 mg per 20 minutes [24]. The higher values in our study are likely due to differences in nicotine concentration in the EC used (16 and 18mg/ml in our study vs 9 mg/ml in the study by Farsalinos et al). Different models of EC (cartomizers vs. tank) were also used in these studies, which could contribute to differences in nicotine delivery. For conventional cigarette smokers, the average nicotine intake is in the range of 0.72–1.16 mg per cigarette [34]. While EC users in our study had a larger range for nicotine intake (0.2–2.6 mg/session), the combined averages for nicotine intake (1.2 mg/10 ml for Blu and 1.4 mg/10 ml for V2) are similar to the upper value of 1.16 mg/cigarette for analog smokers [34].

Compensatory smoking has been reported for users of harm reduction or low-yield cigarettes (e.g., “light” cigarettes) [29, 35–37]. By taking larger puffs, more puffs, puffing more frequently, smoking to a shorter butt length, blocking vent holes, increasing puff velocity, smoking more cigarettes, or removing filters, conventional cigarette smokers have been able to compensate for the lower nicotine delivery in low yield cigarettes [38]. Several lines of evidence suggest that EC users in our study exhibited compensatory puffing. The strongest evidence for compensation is total aerosol intake/10 minutes. Using the ISO standard for conventional cigarettes, an individual who smokes 10 puffs/cigarette every 60 seconds will inhale 350 ml of total smoke in 10 minutes. Based on our data, an EC user who takes 32 puffs (51 ml each) during 10 minutes will take in 1632 ml of aerosol or four times the volume inhaled by the cigarette smoker. The increase in total intake seen with both brands of EC is evidence of compensatory puffing, which would increase the amount of nicotine available for uptake in the oral cavity and lungs. The observation that nicotine intake in EC users was similar to that reported in smokers further supports the idea that compensatory puffing behaviors exist in the EC population.

Some EC users exhibited an interesting “pre-puffing” behavior, indicating a small puff was taken immediately before drawing a larger puff. Pre-puffing may allow EC users to activate or pre-warm the atomizer leading to the delivery of more aerosol and nicotine and consequently a stronger “throat hit”. Generally, users who took pre-puffs had higher puff volumes than the rest of the population, suggesting this is a form of compensatory puffing. To the best of our knowledge, this pre-puffing phenomenon has not been reported for conventional cigarette smokers and may be a new form of compensation characteristic of EC users.

Other factors may facilitate compensatory smoking. For instance, EC can be used repeatedly throughout the day and can easily be smoked for more than 7–10 puffs/session, which is the average number of puffs for one conventional cigarette [4]. This agrees with survey data, in which individuals have reported EC use for more than 20 times/day [39]. In addition, the ease with which puffs can be taken from button-activated EC may facilitate compensatory puffing [5].

While the number of chemicals and their concentrations are lower in EC aerosol than in mainstream smoke from conventional cigarettes [11, 12], some newer models of EC with stronger batteries emit higher concentrations of carbonyl compounds, such as acetaldehyde and formaldehyde, than the original cartridge and cartomizer styles [13]. In addition, some EC emit metals that may cause health problems [40]. These observations coupled with the compensation observed in this study should be factored into actual exposures to chemical toxicants in EC aerosol.

One limitation of the CReSS Pocket may be underestimation of the total EC puff count/10 minutes. Because conventional cigarette smokers do not exceed 43 puffs/per cigarette, the CReSS Pocket is pre-set by the manufacturer to stop recording topography data after a user reaches 43 puffs. In our study, 26% of the user sessions exceeded the CReSS Pocket limit of 43 puff counts. The manufacturer of the CReSS Pocket should correct this feature for future use of the devise with EC. Additionally, it is probable that our average puff duration across both brands (2.65 seconds) is an underestimate due to the exclusion of participants who took large puffs causing fluid to be drawn into the device. In our previous video study, which was not limited by users who took long puffs, average puff duration for EC users was 4.3 seconds [23] and this higher value was confirmed by a second video study [24]. Although these limitations may underestimate the exposure of EC users to nicotine and EC aerosol chemicals, the CReSS Pocket can be validated to collect information on volume/puff, inter-puff interval, flow rate, and peak flow rate, which were not affected by the above limitation.

The adaptation and validation of the CReSS Pocket device for EC topography measurements is new and reliable for most parameters. However, further improvements could be made in the CReSS Pocket for EC topography studies. These would include providing an instruction manual for using the CReSS Pocket with EC, altering the device so that it can record more than 43 puffs/session, and modifying the CReSS so that participants who take long duration puffs can do so without drawing fluid into the mouthpiece. Additionally, the current CReSS Pocket EC adapter would not be suitable for some models of EC (i.e. tank style) that do not resemble conventional cigarettes. Because of the rapid evolution of EC products, it will be important to have topography devices that are adaptable and flexible enough to collect data from various EC styles and designs as they change.

Certain challenges and variables exist when establishing a standard laboratory protocol for production of aerosol from EC. These include: (1) producing a fully adaptable EC topography device for the various styles of EC; (2) accounting for the differences in flow rates needed for the activation of each brand of EC; (3) the varying behaviors of EC users, as well as unique behaviors associated with EC use (i.e., pre-puffing); (4) the significant variation in topography between brands, and (5) understanding that EC topography parameters, such as volume and puff duration, differ from conventional cigarette standards. Due to these challenges and the rapid evolution of EC design and performance, it may be useful to consider creating multiple standard laboratory protocols for EC testing. Factors that should be taken into account when creating EC testing protocols are the differences in performance for various styles of EC [4–6].

Current smoking machine standards for conventional cigarettes vary from each other and from our EC topography data. The International Organization for Standardization (ISO) smoking machine standard (35 ml per 2 second puff every 60 seconds), the Massachusetts standard (45 ml per 2 second puff every 30 seconds) and the Canadian standard (55 ml per 2 second puff every 30 seconds) are uniform in puff duration, but vary in puff volume and puff interval [41]. EC users in our study, on average, took 51 ml puffs of 2.65 seconds duration every 18 seconds. Our data are closest to the Canadian standard, differing mainly in the frequency with which EC users puff (every 18 seconds for EC users vs every 30 seconds for the Canadian standard). Using the best data from the current study and puff duration from Hua et al. (4.3 seconds) and Farsalinos et al. (4.2 seconds) (in which underestimation of puff duration was not a limitation), a reasonable standard for cartomizer style EC would be 51 ml puffs, 4.3 seconds puff duration, at intervals of 18 seconds. These are averages based on the population, and, as for conventional smokers, individual EC values may differ considerably from the average.

In summary, while EC topography is complex and often variable from user to user, individuals often had consistent EC puffing behavior with different brands of EC on different days. The total puff count and intake volume was higher for EC users in a 10-minute period than values reported previously for conventional smokers after one cigarette [29]. Data suggest that intake volumes/10 minutes are greater in dual users than individuals using only EC. Although EC users inhaled on average four times as much aerosol as smokers, nicotine intake for a 10 minute session was similar to that of conventional cigarette users, suggesting EC are inefficient in delivering nicotine. Our data support the idea that EC users participate in compensatory puffing, which has been reported for other harm reduction products with reduced nicotine delivery, such as “light” cigarettes. The high total volume intake by EC users and the emergence of compensatory smoking should be considered in studies of toxicant exposure. Additionally, several participants in the study demonstrated a pre-puffing phenomenon, which was likely adapted to pre-warm the atomizer and optimize nicotine intake. Our data on puff duration [23], puff volume, and puff interval can be used to develop standardized protocols for EC evaluation in research laboratories and clinical studies.
